# Gold Palate Rubber Work

**Published:** 1864-10

**Authors:** Gam’l Jackson

**Affiliations:** Winona


### GOLD PALATE RUBBER WORK.

BY GAM'L JACKSON.

I believe that to Dr. E. Collins is due the credit of first using gold to cover the lingual surface of rubber plates. His method of attaching the plate, as described in the Feb., number of the REGISTER, 1862, consists in punching with a conical punch a row of holes in the plate around its entire circumference, and also around the air chamber. It is easy to understand how the hardened rubber, occupying these holes, which are punched from the concave side of the plate, will hold it firmly in place.

I have been making this class of work ever since I read Dr. Collins' description of it; and rating it the best work yet made from rubber, I will describe my plan of making it, in which it will be seen that the means of attaching the gold plate differs essentially from that already given.

I use gutta percha for trial plates, softening in hot water and rolling on a true surface. A cut from a smooth broom handle may be used as a roller. Strips of annealed sheet brass, or wire passed through the rolling mill until it is reduced to 23, English standard guage plate, should be placed under the ends of the roller so as to secure uniformity of thickness. In adapting the plate to the cast the palatal part should be moulded first, or the plate is liable to be too thin in some places. The posterior edge of the plate should be trimmed to extend a little beyond its finished limits. Having arranged the teeth on the plate as usual and returned it to the cast, the inside wax is trimmed to a level with the pins. The whole lingual surfaces of the plate and teeth being lightly oiled, a cast is taken, using the plaster thick enough to build up without setting over the teeth. The gold plate should be 18 carats and rolled down to about 33. If the arch is high it will be easier struck if a piece is cut out opposite the centrals. Bevel the edges to be joined so as to give a good lap, using fine gold solder for this and other parts of the work. Trim the plate so that when the work is finished there will be a margin of rubber around it one-sixteenth inch wide. The edge of the plate may be finished perfectly square, except on the posterior part, which should be slightly beveled on the concave side. Nine or ten saucer shaped buttons are next to be soldered to the plate about a line from the edge, and it is ready to be adjusted for flasking. In adjusting, it will be necessary to warm the plate so that the buttons will sink into the gutta percha. Wax should now be carefully melted on the border outside the gold plate sufficient for finishing. The buttons are punched from the trimmings of plate. The point of the punch used for this purpose is turned or filed to a broad based truncated cone; and the hole in a small anvil will answer for a counter die. This hole may be 13 of the gauge plate. The buttons should be laid on the file block and touched with a fine file, so that they will set level on the plate. The quantity of solder to each button must be minute; and after soldering, the borax will be removed by boiling in diluted sulphuric acid.

If the rugæ are well defined in the mouth they will be so on the plate, and they, if the arch is high, will be sufficient to hold the plate in place while packing; but if necessary a short piece of wire may be soldered perpendicularly to the middle of the plate. It should be tapered and smooth, as it will be necessary to remove the plate for the purpose of cleansing from gutta percha and wax, for which purpose chloroform or ether may be used. The thickness of the gold plate and button together is 23 of the gauge plate, and the plate when ready for the mouth is 22. An ordinary plate will weigh about three pennyweights.

The several advantages of this style of work are summed up by Dr. Collins, and have been fully realized in my experience with it.

WINONA, Sept., 1864.

