# Erratum

**Published:** 1864-11

**Authors:** 


### ERRATUM.

An omission occurred in my article on "Gold Plate Rubber Work," in the October number of the REGISTER. *There should be six or eight buttons distributed over the plate, besides the row around the border.*

WINONA, MINNESOTA, November, 1864.

